# The Anatomy of the Large American Fluke (*Fasciola Magna*) and a Comparison with Other Species of the Genus Fasciola, S.ST.

**Published:** 1895-05

**Authors:** Chas. Wardell Stiles

**Affiliations:** Zoölogist, Bureau of Animal Industry


### THE ANATOMY OF THE LARGE AMERICAN FLUKE (*FASCIOLA MAGNA*) AND A COMPARISON WITH OTHER SPECIES OF THE GENUS FASCIOLA, S.ST.

BY CHAS. WARDELL STILES, PH.D.,
ZOÖLOGIST, BUREAU OF ANIMAL INDUSTRY.

CONTAINING ALSO A LIST OF THE CHIEF EPIZOÖTICS OF FASCIOLIASIS (DISTOMATOSIS) AND A BIBLIOGRAPHY OF FASCIOLA HEPATICA.

BY ALBERT HASSALL, M.R.C.V.S.,

(Continued from vol. xvi., 1895, page 222.)

### VI. Conclusions.

[These conclusions were printed as a summary of this article: Notes sur les Parasites, 25: La Grande Douve Américaine (*Fasciola magna*); Bul. Soc. Zoöl. d. France, xix., No. 6, séance du 22 Mai (pub. Juin), pp. 91–94.]

The conclusions reached in this paper may be briefly summarized as follows:

1. The generic term *Fasciola* must be retained for flukes of the type of *F. hepatica*.

2. As flukes of this type can be considered *F. magna*, *F. gigantea*, and *F. Jacksoni*.

3. The union of *D. palliatum*, *D. delphini*, *D. Rochebruni*, and *D. oblongum* with the four above-named species of *Fasciola* in a sub-genus of the genus *Distomum* results in the formation of an unnatural and artificial group.

4. Of these four species *D. oblongum* is too poorly described to be taken into consideration; it is probably a *Dicrocœlium* second section.

5. The other three species agree but slightly with *Fasciola*, possessing a somewhat branched intestine, but differing in other very important characters.

6. I therefore propose to follow Cobbold in limiting the genus *Fasciola*[1] (type species *F. hepatica*) and suggest the following as a revised generic diagnosis:

[1] Cf. Règles de la Nomenclature des êtres organisés, Art. 34

*Fasciola* (Linné, pars), type species *F. hepatica*. Hermaphroditic flukes, of the family *Distomidæ*; body large, broad, and more or less leaf-shape (flat), with a more or less distinctly developed anterior conical portion bounded from the broad posterior portion by the ventral acetabulum; œophagus present; intestinal cæca profusely branched; ovary branched, situated for the greater part anterior to transverse vitello-duct; vitellogene glands highly developed and occupying the margins of the posterior portion, ending anteriorly at about the height of the acetabulum; uterus in form of a rosette, anterior to transverse vitello-duct, dorsal and posterior (caudad) to the acetabulum; genital pore about midway between acetabulum and oral sucker; testicles branched, situated for the greater part posterior to transverse vitello-duct; penis present. Oviparous.

Hab. Liver and lungs of herbivorous mammals.

7. The following table will aid in determining the four known species of this genus; the characters relating to *F. Jacksoni* and *F. gigantea* are not satisfactory, but are the only ones which can be taken at present:

| | | | |
|---|---|---|---|
| 1. | Median branches of intestinal cæca[1] long; œsophagus absent; body orbicular, 12 to 16 mm. long by 8 to 12 mm. broad; hab., biliary ducts of Indian elephant . . . | *F. Jacksoni* | p. 144, 1895 |
| | Median branches of intestinal cæca short; body rarely orbicular . | 2 | |
| 2. | Œsophagus[1] extends nearly to acetabulum; body lanceolate, 75 mm. long, 3 to 12 mm. broad; anterior conical portion distinct; posterior extremity obtuse; hab., biliary ducts of giraffe . . . . . . . . . . | *F. gigantea* | p. 139, 1895 |
| | Œsophagus extends half-way to acetabulum or less . . . . | 3 | |
| 3. | Body, flesh-colored, very large and thick, rarely orbicular; 20 to 100 mm. long by 11 to 26 mm. broad; anterior conical portion not very distinct from posterior portion; posterior extremity bluntly rounded; vitellogene glands situated ventrally of the intestine; œsophagus generally one-and-a-half to three times as long as the pharynx; hab., liver and lungs of cattle, deer, etc. . . . . . . . . . . | *F. magna* | p. 172, 1894 |
| | Body, 25 to 35 mm. long by 2 to 4 mm. broad; anterior conical portion much more distinct than in the case of *F. magna*; posterior extremity bluntly pointed; vitellogene gland both dorsal and ventral of intestine; œsophagus rarely one-and-a-half times as long as the pharynx . . . | *F. hepatica* | p. 299, 1894 |

8. If the genus *Fasciola* s.st. is not recognized as distinct from the other distomes, then the generic term *Fasciola* must be used in place of *Distomum*.

[[1] Based upon Cobbold's figures.]

9. As Cobbold failed to state definitely what species he took as the type[1] of the genus *Distomum*, I propose, in order to place the matter beyond doubt, to accept the first species in Cobbold's list, *i. e.*, *Distomum lanceolatum*, as type-species of the genus.

10. As *F. hepatica* was the only species of Dujardin's sub-genus *Cladocœlium*, and hence the type of that sub-genus, the name *Cladocœlium* must fall into synonymy with the separation of *Fasciola* from *Distomum*, and can accordingly not be retained in the genus *Distomum*.

11. *D. palliatum*, *D. delphini*, and *D. Rochebruni*, upon being rejected from the genus *Fasciola*, can be left in the collective genus *Distomum* for the present, but as they differ considerably from the type (*D. lanceolatum*), it is doubtful whether they can remain long in this genus.

12. *F. magna* agrees very closely with *F. hepatica* in the anatomy of the adult, the egg and the miracidium, and it is very probable that the development will be found to be very similar.

13. *F. magna* has a wide distribution in the United States of North America, being very common in certain Southern States (Texas); it has been found also in Northern and Western States (New York, Iowa, Arkansas, Indian Territory, and California); but its frequency in some of these latter States is not determined.

14. It is found in both domesticated and wild animals, sometimes alone, sometimes associated with *F. hepatica*.

15. It has, as Bassi has shown, been introduced into Italy by *Cervus canadensis*, imported from North America.

16. Its close resemblance to *F. hepatica* suggests that it is but a comparatively short time since these two species have become distinct from each other; it is, however, impossible to state whether it existed in this country in wild animals before the discovery of America, and has since become a parasite of domesticated cattle, or whether the parasites were originally introduced with domesticated animals (as *F. hepatica*), and have since then become differentiated into a new species, *F. magna*, which has spread to wild animals.

[1] Cf. Règles de la Nomenclature des êtres organisés, Art. 35 (1889, 1893).

### VII. Compendium of Fasciolas arranged according to their hosts.

A * before a specific name signifies that I have examined specimens of the parasite in question from the host given; the other cases are compiled. Where the specific name of the parasite is in italics, this species is not given for the host in question in von Linstow's *Compendium* or *Nachtrag*. In cases where the parasite is queried (?) the species has been reported for the host in question, but there is probably a mistake in the specific determination.

*Antilope picta* Pallas, 1777, *vide* BOSELAPHUS TRAGOCAMELUS (Pallas), 1767

BOS BUBALIS L. Indian buffalo.
F. hepatica.

BOS TAURUS. Domesticated cattle.
*F. hepatica.
**F. magna.*

BOSELAPHUS TRAGOCAMELUS. Nilgai or blue bull.
F. hepatica.
F. magna.

CAMELUS BACTRIANUS L. Bactrian camel.
F. hepatica.

CAPRA HIRCUS L. Goat.
F. hepatica.

CAVIA COBAYA Schreb. Guinea-pig.
*F. hepatica.*

CARIACUS VIRGINIANUS. Common deer, Virginia deer.
F. hepatica (?).
*F. magna.*

CASTOR FIBER. European beaver.
F. hepatica.

*Cervus aristoteles* Cuv., 1825, *vide* C. UNICOLOR Bechstein, 1799.

CERVUS CANADENSIS. Elk or wapiti.
*F. magna.*

CERVUS DAMA. Fallow deer.
F. hepatica (?).
*F. magna.*

CERVUS ELAPHUS. Stag.
*F. magna.*

CERVUS UNICOLOR. (C. aristoteles). Sanbur, rusa deer.
*F. magna.*

ELEPHAS INDICUS. Indian elephant.
F. hepatica (?).
F. Jacksoni.

EQUUS ASINUS. Ass.
*F. hepatica.

EQUUS CABALLUS. Horse.
*F. hepatica.

FELIS DOMESTICA. Cat.

*F. hepatica.* (*Vide* Stossich '92, p. 8.)

GAZELLA DORCAS. Gazelle.
F. hepatica.

GIRAFFA CAMELOPARDALIS. Giraffe.
F. gigantea.

HOMO SAPIENS. Man.
F. hepatica.

LEPUS CUNICULUS. Wild rabbit.
F. hepatica.

LEPUS CUNICULUS DOM. Domestic rabbit.
F. hepatica.

LEPUS TIMIDUS. Hare.
F. hepatica.

MACROPUS GIGANTEUS. Great gray kangaroo.
F. hepatica.

ORCA GLADIATOR (*vide* p. 302). Grampus "Killer."
**F. hepatica.*

OVIS ARIES L. Sheep.
*F. hepatica.

OVIS ARGALI. BODD. Argali.
F. hepatica.

*Portax picta* Horsfield, *vide* BOSELAPHUS TRAGOCAMELUS (Pallas), 1767.

SCIURUS VULGARIS. European squirrel.
F. hepatica.

SUS SCROFA DOM. Swine.
F. hepatica.

POSTSCRIPT. Since the manuscript of this article was finished over a year ago, several points have come up which it may be well to cover briefly in a postscript.

*Type specimens of* F. gigantea *and* F. Jacksoni. Prof. C. Stewart, of the Royal College of Surgeons of London, writes me that there are no duplicates of these specimens in their museum.

F. hepatica *in Honolulu.* Dr. A. R. Rowat has forwarded specimens of *F. hepatica* from a horse, and in his letter (September 14, 1894) mentions a case of the occurrence of "a fluke" in a woman; only one specimen was passed, details of which were not given.

*The generic term* FASCIOLA. Prof. Max Braun, in reviewing *Notes sur les Parasites*, 25, speaks against my position in using the generic term *Fasciola* in the most emphatic language. As the latter part of this paper appears after Braun's review, a reply may possibly be expected from me by some of my friends. In the review, however, the German helminthologist promises to return to the subject upon the appearance of the present paper, so that any addition to my article upon this score at present would be premature. I will simply state that notwithstanding Braun's views, which, by the way, are simply stated and not supported by arguments, I see no reason for altering my opinions upon this subject as expressed a year ago. That I do not stand alone in the views expressed regarding *Fasciola* is shown by the appearance within a short time of two scientific publications by the French helminthologist, Raphael Blanchard, in which *Fasciola* is adopted.

Stossich (1892, I Distomi degli Ucceli—*the same paper in which his subgenus* Polyorchis, 1888, *is given generic rank, cf. and strike out foot-note p.* 737 *of note* 30) adds *Distomum holostomum* Rud., 1819, and *D. sulcatum* Linston, 1883, two avrian parasites, to the genus *Cladocœlium* (-*Fasciola*). There are no specimens of these parasites in the B. A. I. collection, but judging from the descriptions given I cannot see that they have any generic relationship with *F. hepatica.*

---

The kangaroo is capable of leaping from sixty to seventy feet. The longest recorded leap of a horse is thirty-seven feet.

Cropping dogs' ears is likely to become less common in England, where recently two persons were sent to jail and an owner fined for the offence.

